# An AI-Driven Clinical Decision Support Framework Utilizing Female Sex Hormone Parameters for Surgical Decision Guidance in Uterine Fibroid Management

**DOI:** 10.3390/medicina62010001

**Published:** 2025-12-19

**Authors:** Inci Öz, Ecem E. Yegin, Ali Utku Öz, Engin Ulukaya

**Affiliations:** 1 Department of Gynaecology of Obstetrics, Medicana Atakoy Hospital, Istanbul 34158, Türkiye; 2 Molecular Cancer Research Center, Istinye University, Istanbul 34396, Türkiye; ecem.yegin@istinye.edu.tr (E.E.Y.); eulukaya@istinye.edu.tr (E.U.); 3 Department of Biostatistics and Medical Informatics, Faculty of Medicine, Istinye University, Istanbul 34396, Türkiye; 4 Department of Gynaecology of Obstetrics, Cam & Sakura City Hospital, Istanbul 34480, Türkiye; utkuoz@yahoo.com; 5 Department of Biochemistry, Faculty of Medicine, Istinye University, Istanbul 34396, Türkiye

**Keywords:** uterine fibroid, surgery timing, artificial intelligence, machine learning, female sex hormone

## Abstract

*Background and Objective*: Changes in female sex hormone levels are closely linked to the development and progression of uterine fibroids (UFs). Clinical approaches to fibroid management vary according to guidelines and depend on patient symptoms, fibroid size, and clinician judgment. Despite available diagnostic tools, surgical decisions remain largely subjective. With the advancement of artificial intelligence (AI) and clinical decision support technologies, clinical experience can now be transferred into data-driven computational models trained with hormone-based parameters. To develop a clinical decision support algorithm that predicts surgical necessity for uterine fibroids by integrating fibroid characteristics and female sex hormone levels. *Methods*: This multicenter study included 618 women with UFs who presented to three hospitals; 238 underwent surgery. Statistical analyses and artificial intelligence-based modeling were performed to compare surgical and non-surgical groups. Training was conducted with each hormone—follicle-stimulating hormone (FSH), luteinizing hormone (LH), estrogen (E2), prolactin (PRL), and anti-Müllerian hormone (AMH)—and with 126 input combinations including hormonal and morphological variables. Five supervised learning algorithms—support vector machine, decision tree, random forest, and k-nearest neighbors—were applied, resulting in 630 trained models. In addition to this retrospective development phase, a prospective validation was conducted in which 20 independent clinical cases were evaluated in real time by a gynecologist blinded to both the model predictions and the surgical outcomes. Agreement between the clinician’s assessments and the model outputs was measured. *Results*: FSH, LH, and PRL levels were significantly lower in the surgery group (*p* < 0.001, 0.009, and <0.001, respectively), while E2 and AMH were higher (*p* = 0.012 and 0.001). Fibroid volume was also greater among surgical cases (90.8 cc vs. 73.1 cc, *p* < 0.001). The random forest model using LH, FSH, E2, and AMH achieved the highest accuracy of 91 percent. In the external validation phase, the model’s predictions matched the blinded gynecologist’s decisions in 18 of 20 cases, corresponding to a 90% concordance rate. The two discordant cases were later identified as borderline scenarios with clinically ambiguous surgical indications. *Conclusions*: The decision support algorithm integrating hormonal and fibroid parameters offers an objective and data-driven approach to predicting surgical necessity in women with UFs. Beyond its strong internal performance metrics, the model demonstrated a high level of clinical concordance during external validation, achieving a 90% agreement rate with an independent, blinded gynecologist. This alignment underscores the model’s practical reliability and its potential to reduce subjective variability in surgical decision-making. By providing a reproducible and clinically consistent framework, the proposed AI-based system represents a meaningful advancement toward the validated integration of computational decision tools into routine gynecological practice.

## 1. Introduction

Uterine fibroids (UFs) are benign monoclonal neoplasms originating from the myometrium and represent the most prevalent tumors in women worldwide [1]. The clinical manifestations of UFs commonly include abnormal uterine bleeding leading to anemia, fatigue, chronic vaginal discharge, and dysmenorrhea. Also referred to as leiomyomas, UFs are among the most common benign neoplasms in women. Reported incidence rates vary widely, ranging from 5.4% to 77%, depending on the studied population [2,3]. Treatment options for UFs include nonsteroidal anti-inflammatory drugs, vitamin D3 and iron supplementation, combined oral contraceptives, gonadotropin-releasing hormone (GnRH) analogs, and surgical excision [4,5].

Female sex hormones play a crucial role in fibroid growth and proliferation. This hormonal dependency allows the use of hormone therapy to reduce fibroid size. Hormone-based treatments may be utilized to alleviate symptoms or facilitate preoperative preparation. GnRH agonists inhibit fibroid growth, thereby reducing menstrual bleeding and pain. In cases of menorrhagia-induced anemia, this treatment can also improve hematologic parameters. However, not all patients respond favorably to these hormonal agents; approximately half report minimal or no symptomatic improvement. The suitability of GnRH agonists depends on fibroid type and the planned surgical approach. Several studies have demonstrated their efficacy as preoperative agents prior to UF surgery [6]. Furthermore, GnRH antagonists have recently emerged as promising alternatives for UF management. These agents rapidly bind to GnRH receptors, block endogenous GnRH activity, and directly suppress LH and FSH secretion, thereby avoiding the initial flare-up effect [7].

Clinical management of UFs may vary among specialists depending on existing guidelines, patient symptoms, and clinical findings. With the rapid advancement of artificial intelligence (AI) and clinical decision support systems, these decision-making processes can now be modeled algorithmically, enabling the translation of clinical experience into computational systems [8]. Such systems can assist less-experienced clinicians in making informed and consistent surgical decisions. A critical objective is to develop algorithms that achieve high predictive accuracy. Moreover, designing AI algorithms that operate with minimal input parameters enhances their practicality for routine clinical use.

Vetrivel et al. [9] developed a machine learning-based decision support tool for UF treatment. Using data sourced from Kaggle, the authors trained multiple machine learning (ML) models to predict both treatment decisions and timing. The highest model accuracy achieved was 78%.

Despite the availability of established treatment guidelines, the timing of surgical intervention for UFs remains largely subjective, relying heavily on clinician judgment, symptom interpretation, and patient preference rather than standardized, objective criteria. This variability highlights an unmet need for decision-support tools capable of reducing subjectivity and improving consistency in surgical decision-making. The present study aims to develop a machine learning-based clinical decision support algorithm to guide surgical decision-making for UFs using fibroid characteristics and female sex hormone parameters.

## 2. Materials and Methods

### 2.1. Ethical Consideration

Ethical approval was granted by the Istinye University Ethics Committee (approval date: 30 June 2025; decision number: 24-18). All study procedures adhered to the ethical standards outlined in the Declaration of Helsinki.

### 2.2. Patients

A total of 618 patients diagnosed with UFs who presented to the Departments of Obstetrics and Gynecology at Private Derindere Hospital, Ataköy Medicana Hospital, and Kızılay Kağıthane Hospital (Turkey) were included in the study. Of these, 238 patients underwent surgical intervention. Analyses were planned and conducted by stratifying patients into surgical and non-surgical groups.

Hormonal measurements were obtained as part of routine clinical evaluation and were not standardized to a specific phase of the menstrual cycle. Patients receiving active hormonal therapy at the time of hormone assessment were excluded when this information was clearly documented in the medical records; however, incomplete documentation prevented comprehensive control for prior medical treatments. Surgical intervention was defined based on real-world clinical decision-making by treating physicians rather than predefined guideline-based biological criteria.

### 2.3. Study Design

This study is a national, multicenter, retrospective analysis. Statistical analyses were conducted to examine the relationships between surgical and non-surgical groups across all input parameters. ML training was initially performed for each female sex hormone—follicle-stimulating hormone (FSH), luteinizing hormone (LH), estrogen (E2), prolactin (PRL), and anti-Müllerian hormone (AMH)—independently of UF characteristics. Subsequently, 126 unique input combinations were generated by integrating hormone parameters with UF characteristics, and ML models were trained accordingly. In addition to this retrospective development phase, the model underwent a prospective validation process, during which independent clinical cases were evaluated in real time by a gynecologist blinded to model predictions.

In this study, surgical intervention was defined as the primary outcome variable and was used as a proxy for the real-world clinical decision to operate, rather than an objective biological indication for surgery. Surgical decision-making in uterine fibroid management is inherently multifactorial and may be influenced by symptom severity, treatment refractoriness, fibroid burden, patient preferences, and physician judgment. Because this was a retrospective analysis, detailed and standardized documentation of the specific indications for surgery was not uniformly available across all cases. Accordingly, the developed machine learning models were designed to capture prevailing clinical decision patterns within the observed cohorts, and their outputs should be interpreted as decision-supportive rather than determinative of surgical necessity.

### 2.4. Validation Procedure

To evaluate the external decision-support performance of the developed AI model, an independent validation exercise was conducted using 20 anonymized clinical cases that were not included in the training dataset. These cases were presented to an experienced gynecologist who was blinded to both the model predictions and the surgical outcomes. The clinician was asked to assess each case solely based on the available clinical and hormonal parameters and to determine whether myomectomy was indicated.

For interpretability and consistency, the validating gynecologist was provided exclusively with the same fibroid-related characteristics and female sex hormone parameters used as inputs for the machine learning model. No additional symptom-level information, such as bleeding severity, pain, bulk-related complaints, or patient treatment preferences, was made available during the validation process. This restricted parameter set ensured that the concordance analysis reflected decision alignment based on comparable informational inputs. Inter-rater reliability metrics (e.g., Cohen’s kappa) were not calculated because the validation involved only a single blinded gynecologist.

### 2.5. Statistical Analyses and Tools

Statistical analyses and ML model training were conducted using Wistats v3.0 (WisdomEra Corp., Istanbul, Turkey), incorporating Python-based libraries (SciPy, scikit-learn, statsmodels). Data distribution was assessed via skewness, kurtosis, and the Shapiro–Wilk test. Comparative analyses employed Chi-square or Fisher’s exact tests for categorical variables, and Kruskal–Wallis, one-way ANOVA, *t*-test, or Mann–Whitney U tests for categorical–numerical comparisons. Pearson and Spearman correlations were used for numerical associations. Multivariate logistic regression and other ML algorithms evaluated predictive performance. Statistical hypothesis testing was used for descriptive purposes, whereas the primary analytical focus of the study was the evaluation of multi-input machine learning models trained to capture joint patterns across hormonal and fibroid-related variables under real-world conditions. A *p* value < 0.05 was considered significant.

### 2.6. Machine Learning Procedure and Pipeline

**Figure 1:**
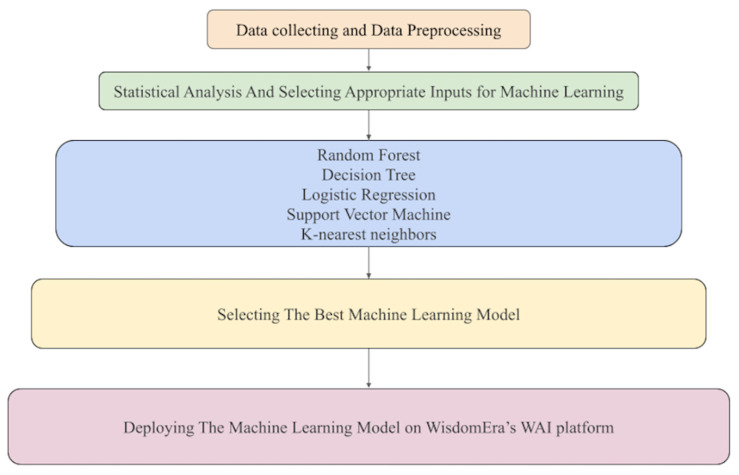
Machine learning procedure and pipeline.

Statistical analyses were conducted prior to ML modeling. Surgical application status was defined as the primary outcome, and predictive algorithms were developed using five classification models: support vector machine (SVM), decision tree (DT), random forest (RF), logistic regression (LR), and k-nearest neighbors (KNN). A 70:30 train-test split was applied for model evaluation, and model performance was evaluated using area under the curve (AUC), accuracy, sensitivity, precision, and F1 score (Figure 1). Five-fold cross-validation was conducted within the training data to obtain a more stable estimate of performance across data partitions.

This study was developed and reported in alignment with emerging methodological frameworks for AI-based clinical prediction models, including principles outlined in TRIPOD-AI [10] and PROBAST-AI [11]. These guidelines emphasize transparent outcome definition, structured model development, rigorous assessment of bias, and clear interpretation of model performance. Although not all components of these evolving frameworks are fully applicable to retrospective datasets, their core principles informed the design, analysis, and reporting of the present work.

Model calibration analyses, including Brier scores or reliability curves, were not conducted in this retrospective dataset. Because calibration assessment requires larger, prospective, and more uniformly documented cohorts, this component is planned for future validation studies. The absence of calibration analysis is acknowledged as a methodological limitation of the present work.

### 2.7. Hyperparameter Configuration

ML models were trained using default hyperparameters without additional tuning. This approach was chosen to prioritize clinical interpretability and to assess whether observed performance reflected intrinsic data structure rather than optimization-driven effects. Model stability was evaluated using cross-validation and external validation procedures.

### 2.8. Class Distribution and Handling of Imbalance

The distribution of surgical classes was moderately imbalanced, with 380 non-surgery cases and 238 surgery cases. No synthetic oversampling or class re-weighting techniques (e.g., SMOTE or cost-sensitive learning) were applied. This decision was intentional, as the primary objective of the study was not to optimize predictive performance under artificially balanced conditions, but rather to model and reproduce real-world clinical classification patterns observed in routine practice.

To mitigate potential bias, training and test splits were generated using stratified sampling to preserve the original class proportions across datasets. Moreover, model performance was evaluated using multiple complementary metrics beyond accuracy, including sensitivity, precision, F1 score, and area under the ROC curve. Finally, external blinded validation served as an additional safeguard against majority-class bias by testing model behavior on independent cases.

### 2.9. Deploying the Decision Support Algorithm in a Web-Based Environment

**Figure 2:**
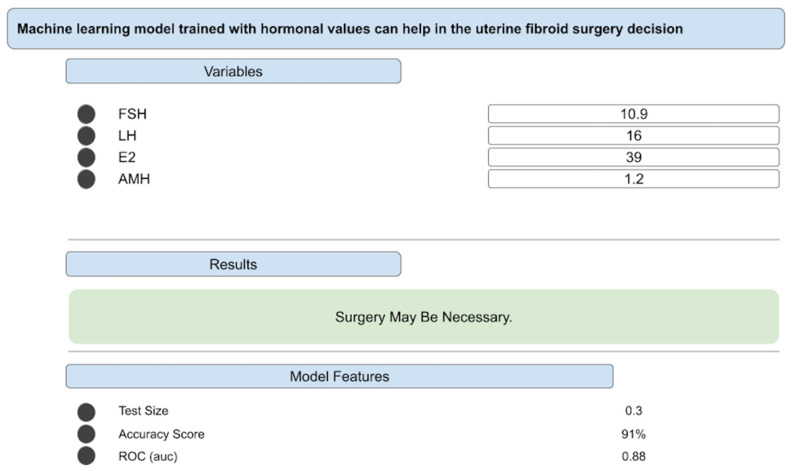
User interface of the deployed machine learning model.

To enable independent reproducibility testing, the highest-performing model was made accessible through a web-based interface (available at JinekoAI.com), which functions solely as an input–output layer without modifying any internal model parameters. The model itself runs on the WisdomEra cloud-based analytics infrastructure (available at WisdomEra.io), which provides the standardized computational backend required for real-time inference. Communication between the interface and the trained model occurs via a secure API, ensuring that predictions are produced exactly as generated by the original machine learning pipeline (Figure 2). This setup allows the model to be evaluated by external users under controlled and reproducible technical conditions while avoiding any form of manual post-training adjustment.

## 3. Results

### 3.1. Statistical Results

**Figure 3:**
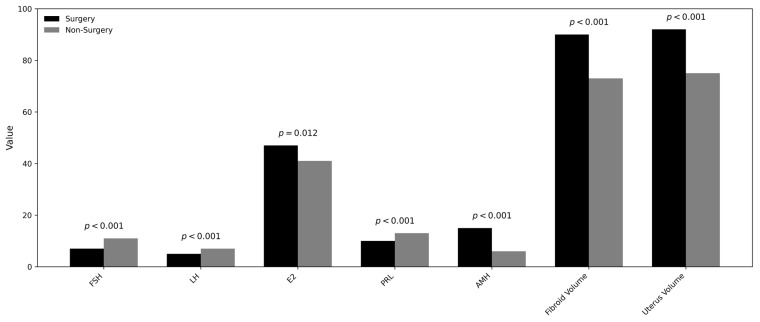
Comparison of hormonal and morphological parameters between surgical and non-surgical groups.

The detailed descriptive and comparative statistics are summarized in Table 1. There was no statistically significant age difference between the surgical and non-surgical groups (*p* = 0.613; mean age: 35.7 vs. 35.4 years). Similarly, the duration of disease (1–5 years vs. >5 years) showed no statistically significant variation between the two groups (*p* = 0.361; proportion of patients with disease > 5 years—surgical: 47%, non-surgical: 43%). In contrast, serum levels of FSH, LH, and PRL were significantly lower in the surgical group than in the non-surgical group (*p* < 0.001, 0.009, and <0.001, respectively). Conversely, E2 and AMH concentrations were significantly higher in the surgical group (*p* = 0.012 and 0.001, respectively). Comparative analysis of UF characteristics revealed that the mean UF volume was markedly greater in the surgical group (*p* <0.001; surgical: 90.8 cc, non-surgical: 73.1 cc). However, the number of UFs did not differ significantly between groups (*p* = 0.384; surgical: 4.7, non-surgical: 4.6).(Figure 3)

### 3.2. Machine Learning Model Training Results

ML models were trained and evaluated to predict surgical necessity by generating 126 input combinations derived from hormonal parameters and UF characteristics. Five different ML algorithms were applied to each input combination, resulting in a total of 630 independent model training sessions. Among these, 12 models achieved an accuracy exceeding 85% and are presented in Table 2. All trained models were categorized according to their accuracy ranges (>90%, 80–90%, 70–80%, 60–70%, 50–60%, and <50%). The distribution of models across these accuracy groups is displayed in Table 3. A complete list of all 126 input combinations and the corresponding performance metrics of the 630 trained models is provided in Supplementary Table S1 to ensure full transparency and reproducibility.

This figure illustrates the complete workflow used to develop the clinical decision support framework. The process begins with data collection and preprocessing, followed by statistical analyses to identify meaningful hormonal and fibroid-related predictors. Five supervised learning algorithms—random forest, decision tree, logistic regression, support vector machine, and k-nearest neighbors—were trained using multiple input combinations. The model with the highest predictive performance was selected and subsequently deployed on the WisdomEra artificial intelligence platform for real-time clinical decision support. This figure demonstrates the stepwise transformation of raw clinical data into a functional and deployable computational tool.

This figure presents the graphical user interface of the implemented clinical decision support tool. The interface allows clinicians to input hormone values—including follicle-stimulating hormone, luteinizing hormone, estradiol, and anti-Müllerian hormone—and instantly receive individualized surgical guidance. The output section displays the model’s decision, such as whether surgical intervention may be necessary. Model performance indicators, including test dataset proportion, accuracy score, and area under the receiver operating characteristic curve, are also provided. This interface demonstrates how the machine learning model is operationalized into a practical decision-support environment intended to enhance clinical workflow and assist clinicians in real-time patient management.

This figure presents a grouped bar chart illustrating mean values of key hormonal markers—follicle-stimulating hormone, luteinizing hormone, estrogen, prolactin, and anti-Müllerian hormone—together with fibroid and uterine volumes in women who underwent surgical intervention versus those managed conservatively. For each parameter, black bars represent the surgical group and gray bars represent the non-surgical group. Corresponding *p*-values above each pair of bars indicate the statistical significance of between-group differences. The chart demonstrates that the surgical cohort exhibited significantly lower follicle-stimulating hormone, luteinizing hormone, and prolactin levels, and significantly higher estrogen and anti-Müllerian hormone levels, as well as larger fibroid and uterine volumes. These trends reflect the combined hormonal and structural features associated with surgical indication in uterine fibroid management.

In multivariable analyses, the most efficient ML model was identified among those utilizing hormonal parameters. Specifically, the RF model trained on LH, FSH, E2, and AMH demonstrated the highest predictive performance, achieving an accuracy of 91%. The test dataset constituted 30% of the total sample. Additional performance metrics were as follows: area under the curve (AUC) = 0.88, Precision = 0.91, Recall = 0.91, and F1-score = 0.91.

When UF volume—a parameter found to differ significantly between surgical and non-surgical groups—was incorporated alongside LH, FSH, E2, and AMH, the resulting model achieved an accuracy of 85%. Using the same 30% test set, its performance metrics were recorded as: AUC = 0.82, Precision = 0.85, Recall = 0.85, and F1-score = 0.85.

### 3.3. External Validation Results

Concordance was observed in 18 of the 20 cases, corresponding to a 90% agreement rate. The two discordant cases were subsequently reviewed and determined to represent borderline clinical scenarios in which surgical decision-making is inherently ambiguous, characterized by intermediate fibroid volumes, non-definitive hormonal profiles, and insufficiently documented symptom severity—factors that historically lead to variability in clinical recommendations. This high level of agreement supports the model’s robustness and demonstrates its potential to align closely with expert clinical judgment in real-world decision-making contexts.

## 4. Discussion

UFs substantially affect women’s health and quality of life, primarily through abnormal or heavy menstrual bleeding and iron deficiency anemia [12]. Making timely and appropriate surgical decisions is therefore critical for optimizing patient outcomes. However, determining the optimal timing of surgery often poses a clinical challenge. The management of UFs should be individualized based on symptom severity and the patient’s reproductive preferences or the desire for definitive treatment [5]. Given the well-established role of female sex hormones in UF pathophysiology [13], our findings demonstrate that hormonal parameters can effectively contribute to clinical decision-support algorithms for predicting surgical necessity. Notably, the RF model incorporating LH, FSH, E2, and AMH achieved the highest predictive accuracy (91%). Furthermore, the UF volume was significantly higher in the surgical group compared with the non-surgical group (*p* < 0.001). Importantly, these model-derived predictions demonstrated strong real-world concordance: in an external validation exercise, the AI model and a blinded gynecologist reached identical surgical decisions in 18 of 20 independent cases, yielding a 90% agreement rate. This level of clinical alignment underscores the model’s capacity to reflect expert decision-making in practice. The observed association between lower FSH and LH levels and higher E2 and AMH concentrations in the surgical group likely reflects a hormonally active, premenopausal endocrine milieu rather than a direct causal mechanism. Premenopausal status is characterized by preserved ovarian reserve and higher estrogen exposure, both of which are known to promote fibroid growth and symptom progression. Elevated AMH levels may serve as a surrogate marker of ovarian reserve and prolonged estrogenic stimulation, potentially contributing to increased fibroid volume and symptom burden that necessitate surgical intervention.

Symptomatic anemia associated with UF progression often influences surgical decision-making. Our results suggest that abnormalities in female sex hormones—particularly decreased FSH and LH levels alongside elevated E2 and AMH—can serve as early predictors of surgical need, even before anemia develops. Increasing UF volume further supports the indication for surgical intervention, particularly in patients with borderline low hemoglobin values. An important and noteworthy finding of this study is that the highest-performing model relied exclusively on female sex hormone parameters without incorporating fibroid volume. This suggests that hormonal profiles may capture upstream biological signals related to fibroid activity and progression that are not fully reflected by static morphological measurements alone. Fibroid volume, while clinically relevant, represents a downstream manifestation of disease burden and may not directly correspond to symptom severity or the timing of surgical decision-making. In contrast, hormonal markers such as FSH, LH, E2, and AMH may provide a more dynamic representation of the endocrine environment driving fibroid growth and symptom evolution. This finding supports the potential utility of hormone-based models as early, objective decision-support tools, particularly in scenarios where fibroid size alone may not fully explain clinical management decisions.

Previous studies have highlighted the hormonal influence on UF growth. Estrogen, LH, and FSH play key regulatory roles in fibroid development [14], and elevated estrogen levels have been linked to accelerated UF growth [15]. Li et al. developed regression-based predictive models identifying FSH, LH, age, and lipid markers as significant predictors of UF growth [13]. In line with these findings, our models utilizing FSH and LH as input features achieved high predictive accuracy (Table 2). We propose that these hormonal parameters can be effectively integrated into ML-based models to estimate surgical necessity in UF patients.

The increasing integration of AI into clinical medicine provides novel opportunities to address diagnostic complexity, personalize treatment planning, and enhance patient outcomes. However, AI-driven decision-support systems remain underutilized in gynecologic practice [16].

Recent studies have demonstrated promising applications of AI in UF diagnosis and treatment. Hue et al. developed an AI-assisted ultrasonography tool that significantly improved diagnostic accuracy among junior ultrasonographers (94.72% vs. 86.63%) compared with senior experts [17]. In our study, we similarly incorporated clinical expertise into AI-based algorithms trained on structured hormonal and UF data. Such models could assist less experienced clinicians in making more informed and timely surgical decisions.

Moreover, other AI applications have shown diagnostic performance comparable to that of human experts in differentiating benign from malignant lesions [18]. Chen et al. reported that AI tools enhanced the precision of hysteroscopic myomectomy by improving spatial localization of submucosal myomas [19].

A recent review by Micić and colleagues summarized current UF management strategies, emphasizing that treatment should begin with medical or minimally invasive approaches before considering surgical intervention. Nonetheless, surgery remains the most common modality. The review also stressed that UF treatment decisions must balance risks and benefits while reflecting patients’ medical profiles and preferences [5]. Consistent with this perspective, our proposed clinical decision-support algorithms offer a systematic, data-driven approach to guide individualized treatment selection.

Surgical intervention is often necessitated by UF-related complications. The AI-based decision-support algorithms developed in this study provide a highly accurate framework for predicting surgical necessity based on hormonal and UF characteristics. These models may enhance decision-making efficiency, reduce inter-clinician variability, and ultimately improve patient outcomes.

From a practical perspective, the proposed AI-based clinical decision-support tool is not intended to replace clinician judgment but to complement it in specific real-world scenarios. The model may be particularly useful as a supportive reference in borderline or equivocal cases where symptom severity, hormonal profile, and fibroid characteristics do not clearly point toward immediate surgical intervention. In such situations, the algorithm can serve as an objective tie-breaker, helping clinicians contextualize hormonal and morphological data alongside clinical judgment. In addition, the tool may have educational value for trainees by illustrating how experienced clinicians integrate hormonal patterns into surgical decision-making. Finally, the model’s transparent, data-driven output may facilitate shared decision-making by helping reassure patients who seek a second opinion or wish to better understand the rationale behind surgical recommendations.

This study has several important limitations that should be carefully considered when interpreting the findings. First, the retrospective design of the primary cohort introduces potential selection and information biases. In addition, the definition of the model’s reference outcome—surgical versus nonsurgical management—does not represent a purely objective biological endpoint but rather reflects a complex clinical decision influenced by symptom severity, patient preferences, and physician-related factors. As a result, there is also a potential risk that the model may perpetuate existing clinical biases embedded in historical surgical decision-making practices rather than correcting them. Postoperative hormone levels were unavailable, preventing assessment of longitudinal hormonal dynamics following surgical intervention. Furthermore, data regarding preoperative pharmacological or interventional treatments were not included, as these variables were not captured in the available database. The external validation, while methodologically rigorous in its blinded design, was limited by a relatively small sample size and should therefore be interpreted as a preliminary concordance analysis rather than definitive clinical validation. Despite these limitations, the ability of the algorithm to approximate surgical decision-making using hormonal and morphological parameters alone demonstrates the feasibility of developing biologically informed, objective decision-support models. Future prospective studies incorporating symptom burden, patient-reported outcomes, treatment preferences, and larger external validation cohorts are warranted to establish clinical impact and facilitate integration into real-world management workflows.

Consistent with emerging recommendations for the development and reporting of AI-based clinical prediction models, including TRIPOD-AI [10] and PROBAST-AI [11], particular emphasis was placed on transparent outcome definition, bias awareness, and cautious interpretation of model performance.

Despite the encouraging external validation results, it is important to acknowledge a central conceptual limitation of this study: the use of surgical intervention as a training label reflects a subjective clinical decision rather than an objective biological endpoint. Consequently, the high concordance observed between the model and the blinded gynecologist may, at least in part, indicate that the algorithm effectively learned prevailing clinical reasoning patterns rather than independently identifying an absolute need for surgery. While this alignment supports the model’s potential utility as a decision-support tool, it also underscores that the system is designed to augment—rather than replace—clinical judgment. This distinction is critical for appropriate interpretation and future clinical integration of AI-driven decision-support algorithms in uterine fibroid management. Currently, no standardized or universally accepted clinical decision rule exists to objectively determine surgical necessity in uterine fibroid management; instead, decisions rely on clinician judgment, symptom burden, fibroid characteristics, and patient preferences. Accordingly, a comparative analysis against an established decision rule was not feasible.

To our knowledge, no prior study has developed an ML-based clinical decision-support algorithm that integrates female sex hormone parameters with UF characteristics to predict surgical necessity. Therefore, this research represents the first such approach reported in the literature and provides a foundation for future AI-driven decision-support systems in gynecology.

## 5. Conclusions

In conclusion, determining the appropriate timing for UF surgery remains a global clinical challenge. The AI-based clinical decision-support algorithms developed in this study can effectively assist in making timely and evidence-based surgical decisions. The high external validation concordance—90% agreement between the model and a blinded gynecologist—demonstrates that the system not only performs well statistically but also aligns closely with real clinical judgment. Our findings underscore the potential of AI to augment clinical expertise, reduce subjective variability, and optimize management strategies for patients with UFs. Further work is underway to refine and expand these AI models for broader surgical decision-making contexts.

## Figures and Tables

**Table 1:** Case characteristics and comparative results between surgery and non-surgery groups.

	Surgery Mean/%	Non-Surgery Mean/%	*p*
Patient *N*, %	238, (38.5)	380, (61.5)	
Age	35.7	35.4	0.613
FSH (mIU/mL)	7.3	10.9	<0.001
LH (mIU/mL)	6.1	7.4	<0.001
E2 (mIU/mL)	47	41.2	0.012
PRL (µg/L)	10.6	13.1	<0.001
AMH (ng/mL)	15.7	6.3	<0.001
Fibroid number	4.7	4.6	0.384
Fibroid volume (cc)	90.8	73.1	<0.001
Uterus volume (cc)	91.9	75.1	<0.001
Disease Duration (years)			0.361
1–5	126 (53)	216 (57)	
>5	112 (47)	164 (43)	

**Table 2:** Machine learning models with an accuracy score greater than 85%.

Inputs	Model	Accuracy	Roc	Precision	Recall	F Score
LH, FSH, E2, AMH	RF	0.91	0.88	0.91	0.91	0.91
LH, FSH, E2, AMH	KNN	0.86	0.84	0.86	0.86	0.86
LH, FSH, PRL, E2, AMH	RF	0.86	0.84	0.86	0.86	0.86
LH, FSH, E2, UF number	RF	0.85	0.82	0.85	0.85	0.85
LH, FSH, E2, UF number	KNN	0.85	0.83	0.85	0.85	0.85
LH, FSH, E2, UF volume	RF	0.85	0.82	0.85	0.85	0.84
LH, FSH, PRL, E2, AMH	KNN	0.85	0.83	0.85	0.85	0.85
LH, FSH, E2, AMH, UF number	RF	0.85	0.82	0.85	0.85	0.85
LH, FSH, E2, AMH, UF number	KNN	0.85	0.83	0.85	0.85	0.85
LH, FSH, E2, AMH, UF volume	RF	0.85	0.82	0.85	0.85	0.85
LH, FSH, E2, UF volume, UF number	RF	0.85	0.81	0.85	0.85	0.84
LH, FSH, PRL, E2, AMH, UF number	RF	0.85	0.82	0.85	0.85	0.85

RF: Random Forest, KNN: K-nearest neighbors, FSH: Follicle stimulating hormone, LH: luteinizing hormone, E2: estrogen, PRL: prolactin, AMH: antimüllerian hormone, UF number: Uterine fibroid number, UF volume: Uterine fibroid volume.

**Table 3:** Number of machine learning models based on accuracy rate groups.

Accuracy Ratio Group	Count
>90%	1
80–90%	136
70–80%	154
60–70%	220
50–60%	117
<50%	2
Total	630

## Data Availability

The dataset generated and analyzed in this study is publicly available on the Istinye University Dataset Sharing Platform. Anonymized clinical and hormonal data related to uterine fibroids can be accessed at the following link: https://dataset.istinye.edu.tr/dataset?did=55 (accessed 11 December 2025). All data were fully anonymized in accordance with ethical regulations. Access is provided for research purposes through a controlled-access system under the platform’s standard licensing and data-sharing policies.

## Supplementary Materials

The following supporting information can be downloaded at: https://www.mdpi.com/article/10.3390/medicina62010001/s1, Table S1: All Machine Learning Results.

## Abbreviations

The following abbreviations are used in this manuscript:

AI	Artificial intelligence
AMH	Anti-Müllerian hormone
AUC	Area under the ROC curve
cc	Cubic centimeter
DT	Decision tree
E2	Estradiol
FSH	Follicle-stimulating hormone
GnRH	Gonadotropin-releasing hormone
KNN	k-nearest neighbors
LH	Luteinizing hormone
LR	Logistic regression
ML	Machine learning
PRL	Prolactin
RF	Random forest
SVM	Support vector machine
UF	Uterine fibroid
UFs	Uterine fibroids
WAI	WisdomEra Artificial Intelligence
